# CT Perfusion in lacunar stroke: a powerful tool to enhance lacunar stroke detection and prediction of patients’ outcome

**DOI:** 10.1007/s10072-026-09262-3

**Published:** 2026-07-31

**Authors:** Emanuele Vincis, Edoardo Ricci, Giovanni Furlanis, Gabriele Prandin, Federica Palacino, Laura Mancinelli, Magda Quagliotto, Michele Malesani, Gianpiero Farina, Paola Caruso, Maja Ukmar, Miloš Ajčević, Marcello Naccarato, Paolo Manganotti

**Affiliations:** 1 Clinical Unit of Neurology, Department of Medicine, Surgery and Health Sciences, Trieste University Hospital ASUGI, University of Trieste, Strada Di Fiume, 447–34149, Trieste, Italy; 2 Radiology Unit, Department of Medicine, Surgery and Health Sciences, Trieste University Hospital ASUGI, University of Trieste, Trieste, Italy; 3 Department of Engineering and Architecture, University of Trieste, Trieste, Italy

**Keywords:** Ischemic stroke, Lacunar stroke, CT perfusion, Stroke prognosis

## Abstract

**Introduction:**

Lacunar strokes are caused by occlusion of small penetrating arteries. Although CT perfusion (CTP) is widely used in the acute settings, automated core/penumbra software often fails to detect lacunar perfusion abnormalities, so its diagnostic and prognostic roles remain unclear. This study aimed to assess the ability of CTP to detect perfusion asymmetries in patients with lacunar stroke and to determine whether such asymmetries are associated with clinical characteristics and functional outcomes.

**Material and methods:**

We retrospectively analyzed 68 patients with lacunar stroke who underwent whole-brain CTP within 4.5 h from stroke onset or recognition. CTP maps (MTT, CBF, CBV, and summary) were visually assessed. Demographic, clinical and imaging data along with stroke metrics and outcomes measurements were collected. Statistical analyses explored CTP detection rate, the association between admission variables and the presence of CTP asymmetries and between these asymmetries and 3-month mRS (dichotomized as either mRS 0–1 or ≥ 2).

**Results:**

41.18% of patients exhibited CTP-detected asymmetries, with the MTT map showing the highest detection rate (39.71%). No demographic or clinical factors predicted CTP positivity. The presence of CTP asymmetries was associated with larger infarct volumes and was independently associated with lower odds of excellent functional recovery (3-month mRS 0–1) (OR 0.232; *p* = 0.012).

**Conclusions:**

Qualitative assessment of individual CTP maps, and especially of MTT map, improves detection of lacunar strokes, particularly with whole-brain coverage. Asymmetries may reflect impaired microvascular perfusion and predict worse outcomes. Therefore, CTP is a valuable diagnostic and prognostic tool in lacunar stroke.

**Supplementary Information:**

The online version contains supplementary material available at 10.1007/s10072-026-09262-3.

## Introduction

Lacunar strokes represent around 25% of all ischemic strokes [1] and typically occurs from the occlusion of a single small penetrating artery [2], with consequent ischemic lesions < 15–20 mm in diameter [3] located in the internal capsule, striatum, corona radiata, thalamus or brainstem. Although lacunar strokes typically arise from small vessel disease caused mainly by arterial hypertension, diabetes mellitus and LDL dyslipidaemia, they can also be caused by embolic events both from cardiac source or due to artery-to-artery embolization, branch atheromatous disease, or rare aetiologies (such as vasculitis or genetic microangiopathies) [4].

Lacunar strokes typically present clinically in the form of the classic lacunar syndromes, i.e. pure hemiparesis or hemiplegia, pure sensory stroke, sensorimotor stroke, ataxic hemiparesis, and dysarthria-clumsy hand syndrome. However Oxfordshire Community Stroke Project Classification (OCSP) [5] alone based on these clinical presentation does not always permit to reliably discriminate between lacunar and small cortical strokes without cortical signs or symptoms [6], making a correlation with neuroimaging mandatory to correctly classify the ischemic event.

Although brain MRI, and in particular diffusion-weighted imaging, is the most sensitive method for the detecting lacunar strokes [7], non-contrast enhanced CT (NCCT), often implemented together with CT angiography (CTA) and CT perfusion (CTP), is the most widely available and commonly employed diagnostic technique worldwide, especially in the emergency setting, since it can be implemented rapidly and costs are generally low [8]. Lacunar perfusion asymmetries are often not identified by post-processed CTP summary core-penumbra map and so are frequently cause of false-negative CTP reports [9], therefore, the utility of CTP in detecting the presence of lacunar stroke in the acute phase remains controversial. In addition, it still needs to be clarified whether CTP may also provide prognostic indications of lacunar stroke patient outcomes.

This paper aims to evaluate the capability of CTP in detecting the presence of ischemic asymmetries in patients with lacunar stroke. We also aimed to investigate whether any clinical factors at presentation could be predictive of the presence of CTP asymmetry and whether the presence of CTP asymmetry correlated with a worse functional outcome.

## Material and methods

### Study population

We retrospectively analyzed demographics, clinical and neuroimaging data of patients of 18 years of age or older presenting with acute-onset neurological deficits consistent with lacunar syndrome compatible with ischemic stroke who underwent CTP evaluation within 4.5 h from stroke onset or within 4.5 h from symptoms recognition in patients with stroke with unknown onset or wake-up stroke and who were subsequently admitted to the Stroke Unit of the Trieste University Hospital (Italy) between September 2020 and December 2022. We excluded patients with Transient Ischemic Attack (TIA), defined as transient neurological deficits lasting less than 24 h [10], patients with neurological deficits due to haemorrhagic stroke or to defined stroke mimics (i.e. patients without evidence of ischemic injury on follow-up brain MRI and evidence of another cause that may explain the presenting symptoms), patients with evidence of vessel occlusion at CTA, patients with cortical hypoperfusion at CTP or cortical ischemic lesion at follow-up CT or MRI scan suggestive of an embolic source of stroke and patients without a CT perfusion imaging or with an inadequate CT perfusion due to technical reasons, such as excessive motion artefacts or suboptimal bolus time.

Recombinant tissue plasminogen activator (rtPA) treatment was offered to all patients who fulfilled the eligibility criteria defined by the ISO-SPREAD VIII edition Italian guidelines for diagnosis and treatment of acute stroke (https://isa-aii.com/linee-guida-spread-viii-edizione/). Patients eligible for thrombolysis were treated with intravenous recombinant tissue plasminogen activator (rtPA), 0.9 mg/kg of body weight, maximum of 90 mg, infused over 60 min with 10% of the total dose administered as an initial intravenous bolus over 1 min.

All patients presented persistent focal neurological deficits after 24 h and/or evidence of cerebral infarction at NCCT follow-up imaging performed 24–48 h after the index event. All patients without evidence of ischemic lesion at the follow-up NCCT underwent brain MRI within 14 days after the ischemic event.

We evaluated all included patients according to a stroke work-up including stroke risk factors assessment, carotid artery ultrasound, electrocardiography, Holter-electrocardiography, and transthoracic echocardiography.

For each patient included in the study, we collected the following data: (1) demographic details (age and sex); (2) established stroke risk factors (atrial fibrillation, type-2 diabetes mellitus, arterial hypertension, dyslipidaemia, smoking, ischemic cardiopathy, history of previous cerebrovascular event); (3) National Institutes of Health Stroke Scale (NIHSS) [11] at baseline and at discharge; (4) pre-admission modified Rankin Scale (mRS) [12], mRS at discharge and 3-month mRS; (5) evidence of early neurological deterioration (END) defined as an elevation of ≥ 2 points on the NIHSS within 72 h of stroke onset [13]; (6) mode of onset (witnessed vs unwitnessed) and time window for IVT (< 4.5 h or unknown onset/wake-up stroke); (7) time from symptoms onset to imaging; (8) lacunar syndrome at admission (i.e. pure hemiparesis or hemiplegia, pure sensory syndrome, sensorimotor syndrome, ataxic hemiparesis, or dysarthria-clumsy hand syndrome); (8) acute treatment administered (IVT); (9) Door to Needle (DTN) time metrics; (10) CTP asymmetries detection rate overall and by single map (summary map, Mean transit time – MTT, Cerebral blood flow – CBF, and CBV – Cerebral blood volume), their volume and their location; (11) presence of ischemic lesions at follow-up NCCT and/or MRI, their volume and their location.

The research was conducted according to the principles of the Declaration of Helsinki.

### NCCT and CTP acquisition and postprocessing

Multiparametric CT protocol involved NCCT, single phase CTA and CTP. All CT imaging was performed with 256-slices CT scanner (Brilliance iCT; Philips Medical Systems, Best, Netherlands). CTP acquisition protocol involves the intravenous injection of 75 mL of contrast medium (Iopromide), followed by a 40 mL saline bolus, both administered at an injection rate of 4 mL/s, and 3-dimensional axial acquisitions on a whole brain volume with a reconstruction of the slices set to 5 mm using a series of repeated movements of the scanner table. Images were acquired every 4 s, resulting in a total scanning time of 60 s. The exposure parameters used were 80 kVp and 150 mAs. CT image processing and analysis were carried out using brain perfusion software (Extended Brilliance Workstation v 3.0, Philips Medical Systems). The perfusion maps MTT, CBV and CBF were obtained after manually positioning a region of interest in correspondence to an artery and a vein. The brain perfusion software is based on the central volume principle and uses a closed-form, noniterative, deconvolution for the evaluation of MTT. The areas below the density/time curves are used to determine the CBV. CBF maps are calculated as a ratio between CBV and MTT. Specific threshold values were set up in the software to identify the penumbra areas (MTT > 7 s or 145% of the contralateral healthy area and CBV > 2.0 mL/100 g) and the infarcted core areas, (MTT > 7 s or 145% of the contralateral healthy area and CBV < 2.0 mL/100 g) as described by Wintermark et al. [14]. Hypoperfusion volumes at individual CTP maps and final ischemic volumes at brain CT or MRI scans were obtained by manual segmentation. All image sets were evaluated by a neuroradiologist.

### Statistical analysis

Statistical analyses were performed using SPSS (Statistical Package for Social Sciences) version 23.0 (SPSS, Chicago, IL). We assessed if the variables followed a normal distribution using the Kolmogorov–Smirnov test. Continuous variables following a normal distribution were presented as mean and standard deviations (SDs), while those with a skewed distribution were presented as median and interquartile ranges (IQRs), indicating the 1 st and 3rd quartile; categorical variables were displayed as counts and percentages (%). Differences between groups were assessed with Student’s t test for continuous variables following a normal distribution, Mann–Whitney U-test for skewed continuous variables, and Pearson’s chi square or exact Fisher test for categorical variables. A *p *value < 0.05 was considered statistically significant. We analyzed the following outcome variables: with regard to the ability of CTP in identifying the presence of a perfusion asymmetry in the acute phase, we analyzed the detection rate of such asymmetries of CTP overall and of each CTP map, visually assessed; with regard to demographic or clinical factors at admission that could predict the presence of asymmetries at CTP, we analyzed the differences in the frequencies of distribution of these factors between patients with CTP with asymmetries and negative CTP; regarding the possible association between the presence of CTP asymmetries and worse functional outcome at follow-up, we analyzed the differences in the frequencies of distribution of different clinical, demographic, and imaging factors in the acute phase among patients with excellent functional independence at 3 months (defined as mRS 0–1 at 3 months). Subsequently, variables with a significance *p* < 0.10 at the univariate analysis as well as patients’ sex were included in univariate and multivariate binary logistic regression models. The results are presented as odds ratios (OR), 95% confidence intervals (95% CI), and *p* values.

Furthermore, a sensitivity analysis was conducted focusing exclusively on the subgroup of patients with radiologically confirmed lesions. This analysis followed the same statistical approach, including univariate comparisons and a subsequent multivariate model, to verify if the prognostic value of CTP asymmetries remained consistent regardless of the eventual visualization of a structural infarct. The results of these model are presented as odds ratios (OR), 95% confidence intervals (95% CI), and *p* values.

## Results

During the study period, of 788 patients admitted to the Stroke Unit of Trieste University Hospital, 148 patients presented with acute-onset neurological deficits consistent with lacunar syndrome compatible with ischemic stroke. 80 patients were subsequently excluded due to the presence of further exclusion criteria, so 68 patients were eligible for the subsequent analyses (as summarized in Supplementary Fig. 1).

Demographic, clinical characteristics at baseline, clinical outcomes as well as stroke characteristics and time metrics are summarized in Supplementary Table 1. The mean age of the included patients was 72 years, with a slight male preponderance of the sample (52.94%). In general, these patients had excellent pre-morbid functionality (median pre-morbid mRS 0), mild-to-moderate stroke on admission (median baseline NIHSS 4), with excellent functional recovery at 3 months in most cases (3-month mRS 0–1 57.35%). In patients with known onset the median time from symptom onset to imaging was 128 min, while 29.41% of patients had unknown onset or wake-up stroke. The most typical lacunar syndrome was pure hemiparesis or hemiplegia (55.88% of cases) and the majority of patients underwent intravenous thrombolysis (58.82%).

To assess potential selection bias, the baseline characteristics of the 49 patients who did not underwent CTP were compared with the 68 included patients (Supplementary Table 2). No major systematic differences were found between the two groups.

CTP and follow-up neuroimaging parameters are summarized in Table 1. Regarding follow-up neuroimaging, the presence of an ischemic lesion at follow-up neuroimaging was detected in 75.00% of all cases (in 11 patients the lesion was identified only by MRI scan), with a median ischemic volume of 0.44 mL and a predominant location in the internal capsule (23.53%).

**Table 1:** **Neuroimaging findings of included patients**

**CTP parameters**	
Presence of perfusion asymmetry at any map	28 (41.18%)
Perfusion asymmetries at each map	
CTP summary map	3 (4.41%)
MTT map	27 (39.71%)
CBV map	18 (26.47%)
CBF map	22 (32.35%)
Volume of perfusion asymmetry	
Volume at CTP summary map	0.49 (0.43–0.87)
Volume at MTT map	0.96 (0.54–1.84)
Volume at CBV map	0.52 (0.32–0.81)
Volume at CBF map	0.72 (0.35–1.13)
Location of perfusion asymmetry	
Internal capsule	11 (16.18%)
Thalamus	5 (7.35%)
Corona radiata	6 (8.82%)
Caudate and lentiform nuclei	2 (2.94%)
Brainstem	4 (5.88%)
**Follow-up imaging parameters**	
Presence of ischemic lesion at F-U imaging	51 (75.00%)
Volume of ischemic lesion at F-U brain imaging	0.44 (0.28–0.98)
Left side of the ischemic lesion at F-U imaging	29 (52.73%)
Location of ischemic lesion at F-U imaging	
Internal capsule	16 (23.53%)
Thalamus	10 (14.71%)
Corona radiata	10 (14.71%)
Caudate and lentiform nuclei	3 (4.41%)
Brainstem	12 (17.65%)

Key: NCCT: Non-contrast-enhanced CT; CTP: CT perfusion; MTT: Mean Transit Time; CBF: Cerebral Blood Flow: CBV: Cerebral Blood Volume; F-U: follow-up

### CTP asymmetries detection rate in patients with lacunar stroke

**Fig. 1:**
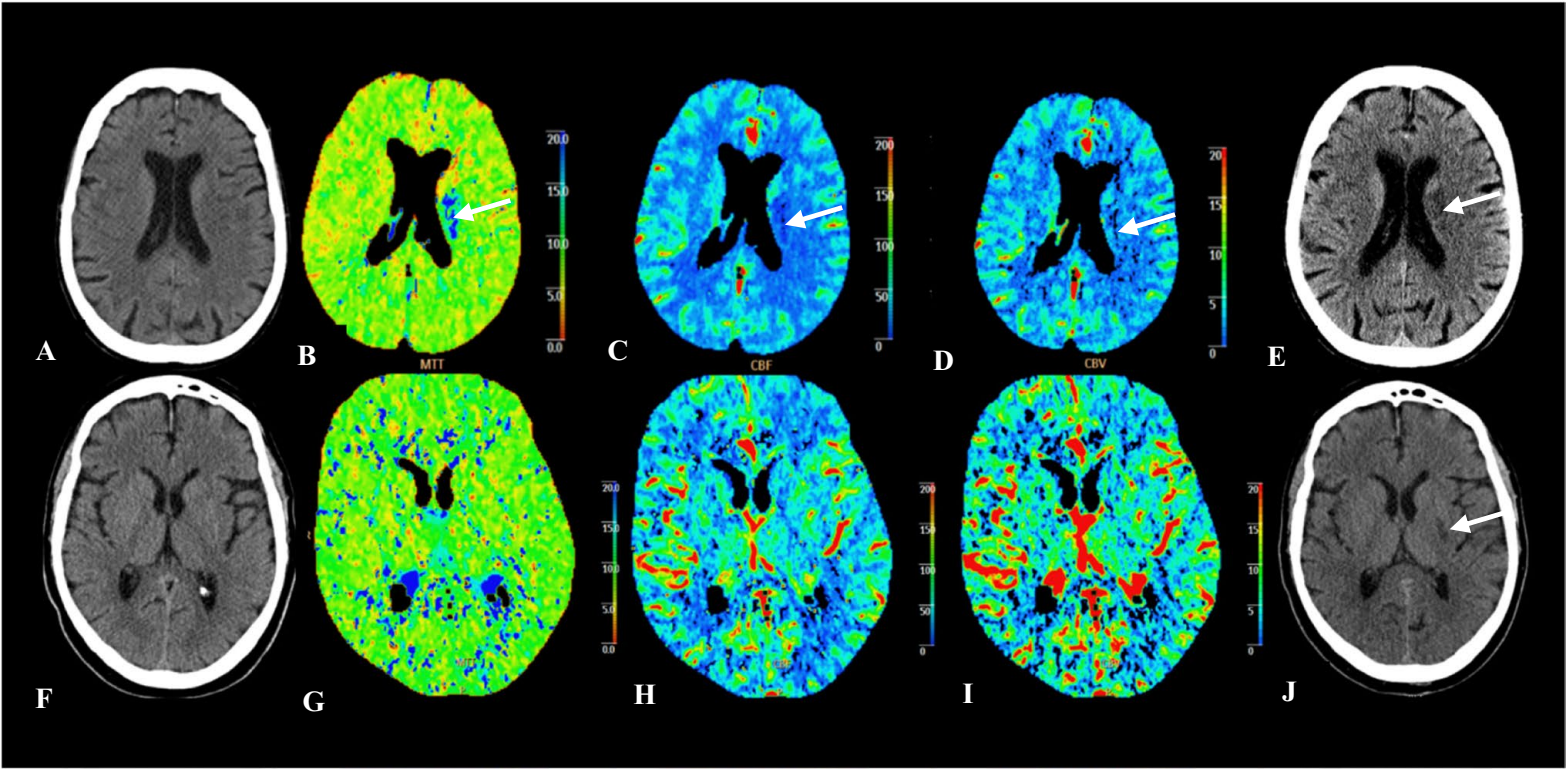
Figures **A**-**E** represent an example of a patient with lacunar stroke with evidence at the individual CTP maps of hypoperfusion (repectively increased MTT at figure **B**, reduced CBF at figure **C**, and reduced CBV at figure **D**), in the absence of visible hypodensity at baseline NCCT but with follow-up NCCT with evidence of ischemic lesion (**E**). Figures **F**-**J**, conversely, represent an example of a patient with lacunar stroke with no evidence of perfusion asymmetry at the CTP maps (**G**, **H**, and **I**), absence of hypodensity at the baseline NCCT (**F**), and presence at the follow-up NCCT of ischemic injury (**J**). White arrows indicate these findings

Of the 68 included patients, 28 (41.18%) presented a perfusion asymmetry in one or more CTP maps while 40 (58.82%) had no asymmetries at CTP maps. So, the overall detection rate of CTP in identifying the presence of perfusion asymmetries in patients with lacunar stroke is found to be 41.18%. The MTT map proved to be the most reliable in identifying such asymmetries, detecting them in 39.71% of cases, followed by the CBF map (detection rate 32.35%), the CBV map (26.47%) and the CTP summary map (4.41%). The most typical location of such asymmetries was the internal capsule (16.18% of patients), with a median volume of hypoperfusion at the MTT map of 0.96 mL. Figure 1 displays an example of lacunar stroke with and without CTP asymmetries.

### Demographic and clinical factors associated with the presence of CTP asymmetries

Comparison of demographic, clinical characteristics at baseline, clinical outcomes as well as stroke characteristics and time metrics between patients with positive CTP and negative CTP is summarized in Table 2. The mean age of patients in the two groups was slightly lower in patients without asymmetry at CTP (70 vs. 76 years), although not statistically significant. There were no significant differences between the two groups regarding sex and cardiovascular risk factors, except for the presence of atrial fibrillation, which was found less frequently in patients with ischemic asymmetry than in patients with CTP without asymmetries (16.18% vs 1.47%, *p* = 0.036).

**Table 2:** **Comparison between demographics and clinical characteristics and outcomes between patients with positive CTP and negative CTP**

	CTP positive (*n* = 28)	CTP negative (*n* = 40)	*p *value
**Age (years)**	76 (± 10)	70 (± 16)	0.065
**Males**	15 (22.06%)	21 (30.88%)	0.931
**Cerebrovascular risk factors**			
Arterial hypertension	21 (30.88%)	31 (45.59%)	0.811
Type 2 diabetes mellitus	8 (11.76%)	11 (16.18%)	0.923
Dyslipidaemia	17 (25.00%)	23 (33.82%)	0.791
Smoke	8 (11.76%)	9 (13.24%)	0.569
Atrial fibrillation	1 (1.47%)	11 (16.18%)	0.036*
Ischemic heart disease	6 (8.82%)	3 (4.41%)	0.146
Previous cerebrovascular event	6 (8.82%)	5 (7.35%)	0.341
**Stroke parameters at admission**			
NIHSS at admission	4 (3–6)	4 (2–5)	0.299
Pre-admission mRS	0 (0–0)	0 (0–0)	0.734
Onset to imaging (if known onset) (minutes)	130 (87–179)	120 (89–177)	0.582
Unknown time of onset/wake-up stroke	9 (13.24%)	11 (16.18%)	0.679
**Lacunar syndrome at admission**			
Pure hemiparesis or hemiplegia	15 (22.06%)	23 (33.82%)	0.748
Pure sensory stroke	0 (0.00%)	1 (1.47%)	1.000
Sensorimotor stroke	6 (8.82%)	6 (8.82%)	0.532
Ataxic hemiparesis	6 (8.82%)	8 (11.76%)	1.000
Dysarthria-clumsy hand syndrome	1 (1.47%)	2 (2.94%)	1.000
**Stroke parameters at discharge and at 3 months**			
NIHSS at discharge	1 (1–4)	1 (0–1)	0.018*
mRS at discharge	3 (1–4)	1 (1–3)	0.059
3-month mRS	2 (1–3)	1 (1–2)	0.038*
3-month mRS 0–1	11 (16.18%)	28 (41.18%)	0.012*
**Reperfusion treatment**			
IVT	15 (22.06%)	25 (36.77%)	0.462
Door to needle (minutes)	60 (52–73)	62 (55–76)	0.718

^*^ Statistically significant data (*p* < 0.05)

Key: NIHSS: National Institutes of Health Stroke Scale; mRS: modified Rankin Scale; IVT: Intravenous thrombolysis

There were no statistically significant differences between the two groups in either pre-event functional independence, severity of neurological deficit at admission, timing from stroke onset to imaging or evidence of an unknown onset stroke or wake-up stroke, lacunar syndrome at admission, or rate and timing of IVT administration. END occurred in only 3 patients with CTP asymmetries and in only 1 patient without CTP asymmetries.

**Fig. 2:**
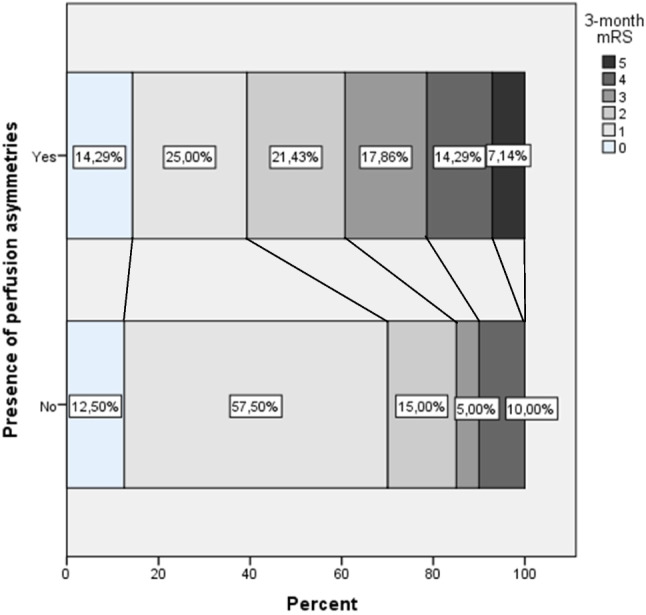
Comparison of 3-month mRS distribution between patients with positive CTP and negative CTP

Patients with CTP asymmetries demonstrated a greater NIHSS compared to patients without CTP asymmetries, a trend in worse mRS at discharge and a worse 3-month mRS (2 vs 1, *p* = 0.038). Figure 2 displays a comparison between the distribution of 3-month mRS of patients with CTP asymmetries and patients without CTP asymmetries.

Comparison between follow-up neuroimaging parameters between patients with CTP positive and CTP negative are summarized in Supplementary Table 3. No differences between groups were detected, with the exception of volume of ischemic lesion at follow-up brain imaging, significantly greater in patients with CTP asymmetries than in patients without asymmetries at CTP (0.65 vs 0.40 mL, *p* = 0.029). Of note, ischemic lesion at follow-up neuroimaging was evident exclusively on brain MRI in 2 patients with positive CTP and in 9 patients with negative CTP.

### Difference in optimal functional outcome in in patients with and without CTP asymmetries

Of the 68 included patients, 39 (57.35%) presented a 3-month mRS score 0–1 while 29 (42.65%) presented a 3-month mRS ≥ 2.

Comparison of demographic, clinical characteristics at baseline, clinical outcomes as well as stroke characteristics and time metrics between patients with 3-month mRS 0–1 and 3-month mRS ≥ 2 are summarized in Table 3. The mean age of patients in the two groups was slightly lower in patients with 3-month mRS 0–1 (76 vs 78 years), although not statistically significant. There were no significant differences between the two groups regarding sex and cardiovascular risk factors, except for the presence of type 2 diabetes mellitus and history of previous cerebrovascular events, which were found less frequently in patients with 3-month mRS score 0–1 compared with patients with 3-month mRS ≥ 2 (respectively, 8.82% vs 19.12%, *p* = 0.008, and 4.41% vs 11.76%, *p* = 0.044).

**Table 3:** **Comparison between demographics and clinical characteristics and outcomes between patients with 3-month mRS 0–1 and 3-month mRS ≥ 2**

	3-month mRS 0–1 (*n* = 39)	3-month mRS ≥ 2 (*n* = 29)	*p *value
**Age (years)**	76 (± 14)	78 (± 14)	0.086
**Males**	20 (29.41%)	16 (23.53%)	0.751
**Cerebrovascular risk factors**			
Arterial hypertension	28 (41.18%)	24 (35.29%)	0.292
Type 2 diabetes mellitus	6 (8.82%)	13 (19.12%)	0.008*
Dyslipidaemia	26 (38.24%)	14 (20.59%)	0.128
Smoke	8 (11.76%)	9 (13.24%)	0.322
Atrial fibrillation	5 (7.35%)	8 (11.76%)	0.126
Ischemic heart disease	3 (4.41%)	6 (8.82%)	0.156
Previous cerebrovascular event	3 (4.41%)	8 (11.76%)	0.044*
**Stroke parameters at admission and at discharge**			
NIHSS at admission	4 (2–5)	4 (3–6)	0.224
Pre-admission mRS	0 (0–0)	0 (0–1)	0.005*
Unknown time of onset/wake-up stroke	12 (17.65%)	8 (11.76%)	0.776
**Lacunar syndrome at admission**			0.643
Pure hemiparesis or hemiplegia	21 (30.88%)	17 (25.00%)	0.695
Pure sensory stroke	1 (1.47%)	0 (0.00%)	1.000
Sensorimotor stroke	6 (8.82%)	6 (8.82%)	0.570
Ataxic hemiparesis	10 (14.71%)	4 (5.88%)	0.364
Dysarthria-clumsy hand syndrome	1 (1.47%)	2 (2.94%)	0.571
**Stroke parameters at discharge and at 3 months**			
NIHSS at discharge	0 (0–1)	2 (1–6)	< 0.001*
mRS at discharge	1 (1–1)	4 (3–4)	< 0.001*
**Reperfusion treatment**			
IVT	25 (36.77%)	15 (22.06%)	0.305
Door to needle (minutes)	63 (55–76)	58 (49–72)	0.479

^*^ Statistically significant data (*p* < 0.05)

Key: NIHSS: National Institutes of Health Stroke Scale; mRS: modified Rankin Scale; IVT: Intravenous thrombolysis

Regarding stroke characteristics and time metrics, patients with 3-month mRS 0–1 presented a pre-morbid mRS lower than patients with 3-month mRS ≥ 2 (*p* = 0.005) as well as a worse NIHSS and mRS at discharge (respectively 0 vs 2, *p* < 0.001, and 1 vs 4, *p* < 0.001). There were no statistically significant differences between the two groups in either severity of neurological deficit at admission, timing from stroke onset to imaging or evidence of an unknown onset stroke or wake-up stroke, lacunar syndrome at admission, or rate and timing of IVT administration.

CTP and follow-up neuroimaging parameters by groups are summarized in Table 4. Regarding CTP parameters, patients with 3-month mRS 0–1 presented a significant lower frequency of CTP asymmetries compared with patients with 3-month mRS ≥ 2 (16.18% vs 25.00%, *p* = 0.011). No differences between groups were found in hypoperfusion volume and location of perfusion asymmetries. Regarding follow-up neuroimaging parameters, no differences between groups were detected, with the exception of volume of ischemic lesion at follow-up brain imaging, significantly greater in patients with 3-month mRS ≥ 2 than in patients 3-month mRS 0–1 (0.68 vs 0.20 mL, *p* < 0.001). Of note, ischemic lesion at follow-up neuroimaging was evident exclusively on brain MRI in 8 patients with 3-month mRS 0–1 and in 3 patients with 3-month mRS ≥ 2.

**Table 4:** **Comparison between neuroimaging findings between patients with 3-month mRS 0–1 and 3-month mRS ≥ 2**

	3-month mRS 0–1 (*n* = 39)	3-month mRS 2–6 (*n* = 29)	*p* value
**CTP parameters**			
Presence of perfusion asymmetry at any map (overall detection rate)	11 (16.18%%)	17 (25.00%)	0.011*
Volume of perfusion asymmetry			
Volume at CTP summary map	1.25 (1.25–1.25)	0.43 (0.39–0.46)	0.667
Volume at MTT map	0.65 (0.54–1.62)	1.20 (0.64–2.30)	0.514
Volume at CBV map	0.40 (0.28–0.68)	0.56 (0.40–0.87)	0.336
Volume at CBF map	0.37 (0.24–0.91)	0.83 (0.42–1.58)	0.127
Location of perfusion asymmetry			
Internal capsule	7 (10.29%)	4 (5.88%)	0.747
Thalamus	1 (1.47%)	4 (5.88%)	0.156
Corona radiata	2 (2.94%)	4 (5.88%)	0.400
Caudate and lentiform nuclei	0 (0.00%)	2 (2.94%)	0.178
Brainstem	1 (1.47%)	3 (4.41%)	0.305
**Follow-up imaging parameters**			
Presence of ischemic lesion at F-U imaging	27 (39.71%)	24 (35.29%)	0.203
Volume of ischemic lesion at F-U brain imaging	0.20 (0.00–0.46)	0.68 (0.40–1.63)	< 0.001*
Left side of the ischemic lesion at F-U imaging	17 (25.00%)	12 (17.65%)	0.722
Location of ischemic lesion at F-U imaging			
Internal capsule	11 (16.18%)	5 (7.35%)	0.292
Thalamus	6 (8.82%)	4 (5.88%)	1.000
Corona radiata	4 (5.88%)	6 (8.82%)	0.305
Caudate and lentiform nuclei	1 (1.47%)	2 (1.47%)	0.571
Brainstem	5 (7.35%)	7 (10.29%)	0.230

^*^ Statistically significant data (*p* < 0.05)

Key: NCCT: Non-contrast-enhanced CT; CTP: CT perfusion; MTT: Mean Transit Time; CBF: Cerebral Blood Flow: CBV: Cerebral Blood Volume; F-U: follow-up

Univariate and multivariate analyses to identify factors already available in the acute phase associated with the frequency of achieving a 3-month mRS 0–1 are displayed in Supplementary Table 4. Variables included in the analyses were age, sex, type 2 diabetes mellitus, history of previous cerebrovascular event, pre-morbid mRS and presence of perfusion asymmetries at CTP. Only CTP asymmetries was found to be an independent factor associated with a lower frequency of 3-month mRS 0–1 at the multivariate analysis (OR 0.248; 95% CI 0.077–0.793; *p* = 0.019).

The same comparison between these groups was performed on the subgroup of 51 patients with a radiologically confirmed ischemic lesion on follow-up. The baseline characteristics and neuroimaging findings of this subgroup are detailed in Supplementary Table 5 and Supplementary Table 6.

Univariate and multivariate analyses to identify factors already available in the acute phase associated with the frequency of achieving a 3-month mRS 0–1 are displayed in Supplementary Table 7. Variables included in the analyses were age, sex, type 2 diabetes mellitus, ischemic heart disease, history of previous cerebrovascular event, pre-morbid mRS and presence of perfusion asymmetries at CTP. The presence of CTP asymmetries was found to be the only independent factor associated with a lower frequency of 3-month mRS 0–1 at the multivariate analysis (OR 0.117; 95% CI 0.022–0.611; *p* = 0.011).

## Discussion

In our study, qualitative analysis of individual whole brain CTP maps allowed identification of a focal alteration in 41.8% of stroke patients presenting with lacunar syndrome. Our data are in line with those of Rudilosso et al. [15], which found an overall CTP sensitivity in the context of lacunar strokes of 50%, using a whole-brain CTP technology like the one employed in our facility. It is of paramount importance, as shown by the results of our study, to qualitatively evaluate each individual map in order to achieve the highest possible detection rate. In fact the detection rate of CTP summary map was found to be only 4.41%. This fact is strongly supported by a recent systematic review and from Zedde et al. [9] and by various observational studies [16–18], such as that of Garcia-Esperon et al., which found an overall sensitivity of 42.4% by visually assessing each CTP map vs a sensitivity of only 5.9% by the automated core/penumbra software [18]. Moreover, while a quantitative ROI-based or voxel-based analysis could theoretically provide more objective data, such methods are technically challenging in lacunar stroke due to the small lesion size and the significant impact of partial volume effects and physiological noise in the deep brain structures. The use of whole brain CT as in our study has been shown to increase the detection rate of perfusion asymmetries, especially in low-flow regions such as the brainstem, thus aiding in the identification of lacunar strokes at that site as well [9, 15, 19]. In literature, time-based maps seem to be more sensitive than flow-based maps [9, 20]. In particular, we demonstrated that the MTT map is the most reliable in correctly identifying the presence of perfusion asymmetries in this clinical context, being positive in 39.71% of patients. Similar detection rate values of the MTT map (around 30–40%) have also been demonstrated in the work of Esperon-Garcia et al. [18] and Benson et al. [17]. The data from Cao et al. [16] showed even higher sensitivity values for this map (56.7%), but it must be considered that the sensitivity analysis was only performed on patients with a DWI-confirmed ischemic lesion. The superior predictive ability of MTT map compared to CBF and CBV probably derives from its high sensitivity to subtle prolonged transit times. In the microvascular territory of penetrating arteries, MTT is the first parameter to alter when perfusion pressure drops, while CBF and CBV may remain relatively compensated through auto-regulatory mechanisms. Thus MTT map seems to effectively capture the underlying hemodynamic failure, making it the most reliable predictor of the final infarct extent and subsequent clinical outcome [21]. It should be noted, however, that the group of Rudilosso et al. [15] showed a higher sensitivity of the time-to-drain (TTD) map, which is not available in our institution. Additionally, also Tmax map was not available at our institution during the study period. However, while acknowledging that Tmax is the most widely used parameter for detecting areas of hypoperfusion in acute ischemic stroke, current literature suggests that the pathophysiological profile of lacunar stroke may be better captured by MTT than by Tmax. In the microvascular territory of small penetrating arteries, perfusion disturbances are often subtle. MTT has been shown to offer superior sensitivity in visually detecting early prolongations in transit time that might not be as conspicuous on Tmax maps [9–21].

It is noteworthy that the ghost core phenomenon represents a potential confounder in the hyper-acute phase, when initial perfusion alterations can occasionally overestimate the definitive infarct size due to transient hypoperfusion that may resolve before tissue death. While this phenomenon is primarily described in major vascular territories because of a collateral failure, it warrants caution when interpreting CTP maps in the hyperacute stage of lacunar syndromes as well [22, 23].

No single demographic or clinical factor at admission was able to predict the presence of CTP asymmetries, although there was a trend towards a lower frequency of CTP asymmetries in patients with atrial fibrillation, which was not confirmed by binary logistic regression. This finding should be investigated in future work, since, for other types of stroke, the presence of atrial fibrillation has been shown to be associated with an increased frequency of CTP asymmetries, as recently highlighted in a paper on posterior circulation strokes [24]. Nor was the time from symptom onset to imaging associated with evidence of perfusion asymmetries at CTP.

Recently, it has been observed that although the traditional penumbra/core hypothesis, which assumes the presence of an area of irreversible hypoperfusion (core) and an area with viable tissue (penumbra) enabled by the presence of collateral circulation, has long been considered not applicable to lacunar strokes, this assumption may not be entirely correct [25]. In fact, post-mortem [26] and advanced neuroimaging studies [27] have shown that the perforating arteries are not terminal vessels completely devoid of a collateral circulation, but rather that retrograde and anterograde collateral circulations exist, which, if functional, can maintain the parenchyma vascularized by the perforating arteries viable despite the occlusion of the perforating arteries themselves. Such evidence may account for the fact that patients with lacunar stroke and hypoperfusion at CTP present a larger ischemic lesion at follow-up neuroimaging, as shown by our study and by the work of Huang et al. [27], and an increased incidence of early neurological deterioration (END), probably because of the impairment of the collateral circulation in maintaining parenchymal perfusion. This relationship between the presence of END and the presence of CTP asymmetries has been demonstrated in several studies [4, 13, 14]. Our multivariate analysis evidenced that CTP status is an independent predictor of optimal functional outcome at 3 months, in particular highlighting how patients with CTP asymmetries demonstrated lower odds of achieving a 3-month mRS 0–1 compared to patients without CTP alteration, with an OR 0.248 (95% CI 0.077–0.793). This remained true also for the subgroup of patients with a radiological confirmation of the ischemic lesion at follow-up neuroimaging, where the presence of CTP asymmetry strongly predicted lower odds of achieving a 3-month mRS 0–1 compared to patients without CTP alterations, with an OR 0.117 (95% CI 0.022–0.611). Noteworthy, the lack of follow-up lesions in 25% of the total cohort likely reflects a combination of clinical and pathophysiological factors. First, the impact of reperfusion therapies is significant; in our cohort, 58.8% of patients received intravenous thrombolysis. In several instances, the treatment likely resolved the hemodynamic distress captured by the CTP before it could evolve into permanent tissue necrosis, effectively "saving" the tissue [28]. Second, the timing of the MRI is crucial since many patients underwent NCCT at 24 h and MRI at 48 h (since the symptoms were mild and patients were to be discharged at home as soon as possible), a window in which DWI sensitivity for millimetric lacunar lesions is known to be imperfect, especially in the brainstem locations [29]. However we acknowledge that this subgroup might include cases of radiological TIA (as defined by absence of ischemic infarct at follow-up neuroimaging), despite objective clinical deficits lasting more than 24 h (consistent with the common definition of TIA, endorsed also by the ESO) [10] but without a visible structural correlates on imaging.

Despite these considerations, since the prognostic value of CTP has been demonstrated both in the overall cohort and within the strictly radiologically-confirmed subgroup, this imaging modality remains a powerful and reliable indicator of clinical outcome. This suggests that the early hemodynamic impairment captured by CTP, and particularly by MTT map, provides long-term prognostic information that exceeds the baseline clinical assessment, identifying patients who may require more intensive secondary prevention or closer follow-up, reinforcing the usefulness of CTP to provide prognostic information as well as an important tool to help the diagnosis in the acute setting [20, 30–32].

### Strengths and limitations

Our study has several limitations, such as its monocentric nature, the relatively small number of patients enrolled, a cohort composed solely of individuals of Caucasian descent, the adoption of a single CTP evaluation software as well as the lack of analysis of other CTP maps (in particular TTD and Tmax) and the visual assessment of CTP maps was performed by a single neuroradiologist, which introduces a potential risk of inter-observer bias and may limit the generalizability of our findings. Moreover the retrospective nature of this study represents a significant limitation. While it reflects real-world clinical practice in an emergency setting, this design is inherently susceptible to selection bias and potential confounding factors. Consequently, our results should be interpreted with caution, and further prospective, multicenter studies are required to confirm these findings. Finally, the lack of radiological confirmation in 25% of our cohort represents a limitation and, despite reflecting the real-world scenario with potential tissue-saving effect of intravenous thrombolysis and the imperfect sensitivity limits of MRI in brainstem circulation stroke, it may have introduced some degree of heterogeneity. The main strengths of our study are that it reinforces the body of evidence that CTP is a powerful tool to enhance lacunar stroke detection in the hyperacute phase and it is the first study, to the best of our knowledge, to demonstrate the association between CTP asymmetries, a variable already available in the hype-acute setting, and a lower odds of achieving optimal functional independence at follow-up (i.e. 3-month mRS 0–1) in patients with lacunar stroke syndrome, in both patients with and without evidence of ischemic lesion at follow-up neuroimaging.

## Conclusion

Our study supports the usefulness of qualitative analysis of each CTP map in order to increase the detection rate of ischemic asymmetries, even in patients with lacunar stroke, traditionally considered inefficiently detectable by acute-phase CT methods. We also demonstrated how CTP is also a relevant prognostic tool, showing a lower probability of achieving an excellent functional outcome at 3 months in patients with lacunar stroke and CTP asymmetries.

## Supplementary Information

Below is the link to the electronic supplementary material.

Supplementary file1 (DOCX 34 KB)

Supplementary file2 (DOCX 1936 KB)

## Data Availability

The data will only be made available from the corresponding author upon reasonable request.
