# Supplementary material for: CT Perfusion in lacunar stroke: a powerful tool to enhance lacunar stroke detection and prediction of patients’ outcome
Source: Neurol Sci. 2026 Jul 31;47(8):671. doi: 10.1007/s10072-026-09262-3 (PMC13423970; doi:10.1007/s10072-026-09262-3)
Supplement: Supplementary file 1 — Supplementary file1 (DOCX 34 KB) [file 10072_2026_9262_MOESM1_ESM.docx]

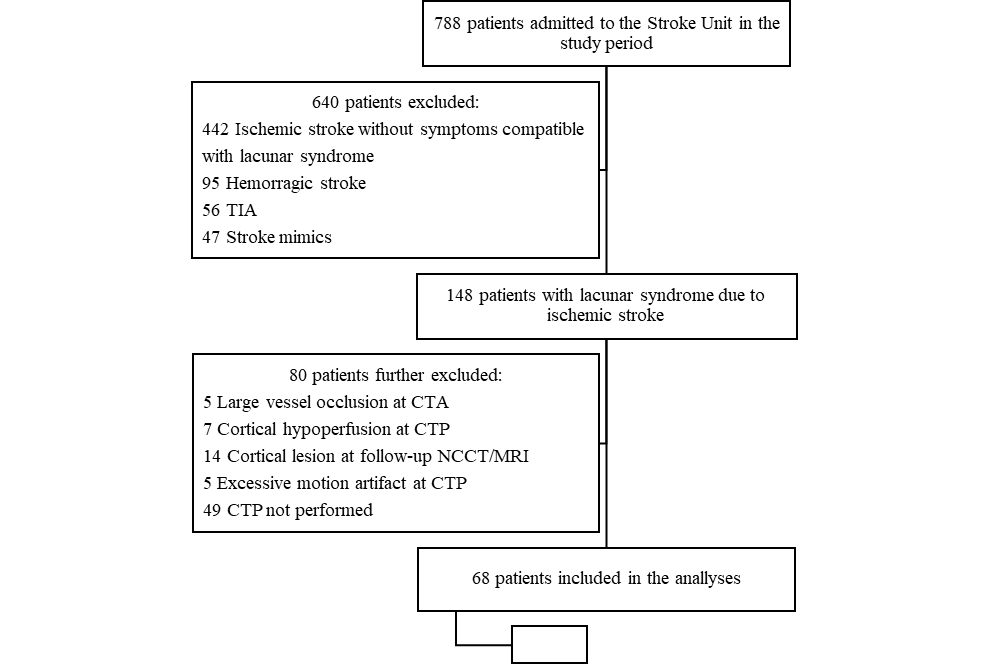


Supplementary Figure 1: Flow chart describing the selection of patients included in the analyses.
