# Supplementary material for: CT Perfusion in lacunar stroke: a powerful tool to enhance lacunar stroke detection and prediction of patients’ outcome
Source: Neurol Sci. 2026 Jul 31;47(8):671. doi: 10.1007/s10072-026-09262-3 (PMC13423970; doi:10.1007/s10072-026-09262-3)
Supplement: Supplementary file 2 — Supplementary file2 (DOCX 1936 KB) [file 10072_2026_9262_MOESM2_ESM.docx]

| **Supplementary Table 1 –Demographics and clinical characteristics of included patients** | |
| --- | --- |
| **Age** (years)  **Males** | 72 (±14)  36 (52.94%) |
| **Cerebrovascular risk factors** |  |
| Arterial hypertension | 52 (76.47%) |
| Type 2 diabetes mellitus | 19 (27.94%) |
| Dyslipidaemia | 40 (58.82%) |
| Smoke | 17 (25.00%) |
| Atrial fibrillation | 12 (17.65%) |
| Ischemic heart disease | 9 (13.24%) |
| Previous cerebrovascular event | 11 (16.18%) |
| **Stroke parameters at admission** |  |
| NIHSS at admission | 4 (3-5) |
| Pre-admission mRS | 0 (0-0) |
| Onset to imaging (if known onset) (minutes) | 128 (88-179) |
| Unknown time of onset / wake-up stroke | 20 (29.41%) |
| **Lacunar syndrome at admission** |  |
| Pure hemiparesis or hemiplegia | 38 (55.88%) |
| Pure sensory stroke | 1 (1.47%) |
| Sensorimotor stroke | 12 (17.65%) |
| Ataxic hemiparesis | 14 (20.59%) |
| Dysarthria-clumsy hand syndrome | 3 (4.41%) |
| **Stroke parameters at discharge and at 3 months** |  |
| NIHSS at discharge | 1 (0-2) |
| mRS at discharge | 2 (1-4) |
| 3-month mRS | 1 (1-2) |
| 3-month mRS 0-1 | 39 (57.35%) |
| **Reperfusion treatment** |  |
| IVT | 40 (58.82%) |
| Door to needle (minutes) | 61 (54-77) |
|  |  |
| Key: NIHSS: National Institutes of Health Stroke Scale; mRS: modified Rankin Scale; IVT: Intravenous thrombolysis. | |

| **Supplementary Table 2 – Comparison of demographics and clinical characteristics of included patients vs patients who did not underwent CTP** | | | |  |  |
| --- | --- | --- | --- | --- | --- |
|  | **Included patients (n=68)** | **Patients who did not underwent CTP (n=49)** | ***p value*** | |  |
| **Age** (years)  **Males** | 72 (±14)  36 (52.94%) | 71 (±10)  28 (57.14%) | 0.639  0.851 | |  |
| **Cerebrovascular risk factors** |  |  |  | |  |
| Arterial hypertension | 52 (76.47%) | 43 (87.75%) | 0.123 | |  |
| Type 2 diabetes mellitus | 19 (27.94%) | 14 (28.57%) | 0.940 | |  |
| Dyslipidaemia | 40 (58.82%) | 34 (69.39%) | 0.242 | |  |
| Smoke | 17 (25.00%) | 13 (26.53%) | 0.852 | |  |
| Atrial fibrillation | 12 (17.65%) | 5 (10.20%) | 0.260 | |  |
| Ischemic heart disease | 9 (13.24%) | 4 (8.16%) | 0.389 | |  |
| Previous cerebrovascular event | 11 (16.18%) | 8 (16.33%) | 0.983 | |  |
| **Stroke parameters at admission** |  |  |  | |  |
| NIHSS at admission | 4 (3-5) | 5 (3-6) | 0.480 | |  |
| Pre-admission mRS | 0 (0-0) | 0 (0-0) | 0.986 | |  |
| Key: NIHSS: National Institutes of Health Stroke Scale; mRS: modified Rankin Scale. | | | | | |

| **Supplementary Table 3 – Comparison between neuroimaging findings between patients with positive CTP and negative CTP** | | | |
| --- | --- | --- | --- |
|  | **CTP positive (n = 28)** | **CTP negative (n = 40)** | ***p value*** |
| **Follow-up imaging parameters** |  |  |  |
| Presence of ischemic lesion at F-U imaging | 24 (35.29%) | 27 (39.71%) | 0.088 |
| Volume of ischemic lesion at F-U imaging | 0.65 (0.34-1.63) | 0.40 (0.23-0.62) | 0.029* |
| Left side of the ischemic lesion at F-U imaging | 16 (23.53%) | 13 (19.12%) | 0.341 |
| Location of ischemic lesion at F-U imaging |  |  |  |
| Internal capsule | 9 (13.24%) | 7 (10.29%) | 0.161 |
| Thalamus | 4 (5.88%) | 6 (8.82%) | 1.000 |
| Corona radiata | 6 (8.82%) | 4 (5.88%) | 0.297 |
| Caudate and lentiform nuclei | 2 (1.47%) | 1 (1.47%) | 0.564 |
| Brainstem | 3 (4.41%) | 9 (13.24%) | 0.334 |
| * Statistically significant data (p < 0.05).  Key: NCCT: Non-contrast-enhanced CT; CTP: CT perfusion; MTT: Mean Transit Time; CBF: Cerebral Blood Flow: CBV: Cerebral Blood Volume; F-U: follow-up. | | | |

| **Supplementary Table 4 – Univariate and multivariate binary logistic regression analysis to identify factors available in the acute phase associated with the frequency of achieving a 3-month mRS 0-1** | | | | |
| --- | --- | --- | --- | --- |
|  | Univariate analysis | | Multivariate analysis | |
|  | OR (95% CI) | *p* value | OR (95% CI) | *p* value |
| Age | 0.968 (0.931-1.006) | 0.094 | 0.989 (0.945-1.034) | 0.620 |
| Male sex | 1.169 (0.446-3.067) | 0.751 | 1.207 (0.387-3.764) | 0.745 |
| Type 2 diabetes mellitus | 0.224 (0.072-0.697) | 0.069 | 0.294 (0.079-1.099) | 0.069 |
| Previous cerebrovascular event | 0.219 (0.052-0.916) | 0.037* | 0.322 (0.059-1.740) | 0.188 |
| Pre-admission mRS | 0.373 (0.148-0.939) | 0.036* | 0.459 (0.167-1.259) | 0.130 |
| Presence of perfusion asymmetry at any map | 0.277 (0.100-0.766) | 0.013* | 0.248 (0.077-0.793) | 0.019* |
|  |  |  |  |  |
| * Statistically significant data (p < 0.05).  Key: mRS: modified Rankin Scale. | | | | |

| **Supplementary Table 5 – Comparison between demographics and clinical characteristics and outcomes between patients with 3-month mRS 0-1 and 3-month mRS ≥2 (only patients with ischemic lesion at follow-up)** | | | |
| --- | --- | --- | --- |
|  | **3-month mRS 0-1 (n = 27)** | **3-month mRS ≥ 2 (n = 24)** | ***p value*** |
| **Age** (years) | 66 (±15) | 73 (±14) | 0.065 |
| **Males** | 13 (48.1%) | 12 (50.0%) | 1.000 |
| **Cerebrovascular risk factors** |  |  |  |
| Arterial hypertension | 18 (66.7%) | 19 (79.2%) | 0.363 |
| Type 2 diabetes mellitus | 4 (14.8%) | 10 (41.7%) | 0.058 |
| Dyslipidaemia | 17 (63.0%) | 12 (50.0%) | 0.405 |
| Smoke | 7 (25.9%) | 9 (37.5%) | 0.546 |
| Atrial fibrillation | 3 (11.1%) | 5 (20.8%) | 0.451 |
| Ischemic heart disease | 1 (3.7%) | 5 (20.8%) | 0.088 |
| Previous cerebrovascular event | 2 (7.4%) | 6 (25.0%) | 0.090 |
| **Stroke parameters at admission and at discharge** |  |  |  |
| NIHSS at admission | 4 (2-5) | 4 (3-6) | 0.190 |
| Pre-admission mRS | 0 (0-0) | 0 (0-1) | 0.098 |
| Unknown time of onset / wake-up stroke | 10 (37.0%) | 7 (29.2%) | 0.767 |
| **Lacunar syndrome at admission** |  |  | 0.983 |
| Pure hemiparesis or hemiplegia | 15 (55.6%) | 13 (54.2%) | 1.000 |
| Pure sensory stroke | 1 (3.7%) | 0 (0.0%) | 1.000 |
| Sensorimotor stroke | 5 (18.5%) | 5 (20.8%) | 1.000 |
| Ataxic hemiparesis | 5 (18.5%) | 4 (16.7%) | 1.000 |
| Dysarthria-clumsy hand syndrome | 1 (3.7%) | 2 (8.3%) | 0.596 |
| **Stroke parameters at discharge and at 3 months** |  |  |  |
| NIHSS at discharge | 0 (0-1) | 3 (1-7) | <0.001* |
| mRS at discharge | 1 (1-1) | 4 (3-4) | <0.001* |
| **Reperfusion treatment** |  |  |  |
| IVT | 15 (55.6%) | 12 (50%) | 0.782 |
| Door to needle (minutes) | 65 (52-79) | 59 (48-74) | 0.432 |
|  |  |  |  |
| * Statistically significant data (p < 0.05).  Key: NIHSS: National Institutes of Health Stroke Scale; mRS: modified Rankin Scale; IVT: Intravenous thrombolysis. | | | |
|  |  |  |  |

| **Summary Table 6 – Comparison between neuroimaging findings between patients with 3-month mRS 0-1 and ≥2**  **(only patients with ischemic lesion at follow-up)** | | | |
| --- | --- | --- | --- |
|  | **3-month mRS 0-1 (n = 27)** | **3-month mRS 2-6 (n = 24)** | ***p value*** |
| **CTP parameters** |  |  |  |
| Presence of perfusion asymmetry at any map (overall detection rate) | 7 (25.9%) | 17 (70.8%) | 0.002* |
| Volume of perfusion asymmetry |  |  |  |
| Volume at CTP summary map | 1.25 (1.25-1.25) | 0.45 (0.36-0.51) | 1.000 |
| Volume at MTT map | 0.51 (0.44-1.35) | 1.08 (0.50-2.20) | 0.354 |
| Volume at CBV map | 0.28 (0.23-0.62) | 0.56 (0.39-0.95) | 0.130 |
| Volume at CBF map | 0.24 (0.18-1.24) | 0.83 (0.42-1.73) | 0.101 |
| Location of perfusion asymmetry |  |  | 0.002* |
| Internal capsule | 5 (18.5%) | 4 (16.7%) | 1.000 |
| Thalamus | 0 (0.0%) | 4 (16.7%) | 0.042* |
| Corona radiata | 2 (7.4%) | 4 (16.7%) | 0.402 |
| Caudate and lentiform nuclei | 0 (0.0%) | 2 (8.3%) | 0.217 |
| Brainstem | 0 (0.0%) | 3 (12.5%) | 0.097 |
| **Follow-up imaging parameters** |  |  |  |
| Volume of ischemic lesion at F-U brain imaging | 0.44 (0.30-0.54) | 1.25 (0.40-1.86) | 0.002 |
| Left side of the ischemic lesion at F-U imaging | 15 (55.6%) | 12 (50.0%) | 0.782 |
| Location of ischemic lesion at F-U imaging |  |  | 0.489 |
| Internal capsule | 11 (40.7%) | 5 (20.8%) | 0.145 |
| Thalamus | 6 (22.2%) | 4 (16.7%) | 0.732 |
| Corona radiata | 4 (14.8%) | 6 (25.0%) | 0.485 |
| Caudate and lentiform nuclei | 1 (3.7%) | 2 (8.3%) | 0.596 |
| Brainstem | 5 (18.5%) | 7 (29.2%) | 0.511 |
|  |  |  |  |
| * Statistically significant data (p < 0.05).  Key: NCCT: Non-contrast-enhanced CT; CTP: CT perfusion; MTT: Mean Transit Time; CBF: Cerebral Blood Flow: CBV: Cerebral Blood Volume; F-U: follow-up. | | | |

| **Supplementary Table 7 – Univariate and multivariate binary logistic regression analysis to identify factors available in the acute phase associated with the frequency of achieving a 3-month mRS 0-1 (only patients with ischemic lesion at follow-up)** | | | | |
| --- | --- | --- | --- | --- |
|  | Univariate analysis | | Multivariate analysis | |
|  | OR (95% CI) | *p* value | OR (95% CI) | *p* value |
| Age | 0.964 (0.924-1.005) | 0.084 | 1.006 (0.955-1.059) | 0.834 |
| Male sex | 1.077 (0.358-3.235) | 0.985 | 0.655 (0.156-2.747) | 0.563 |
| Type 2 diabetes mellitus | 0.243 (0.064-0.926) | 0.038* | 0.167 (0.028-0.998) | 0.050 |
| Ischemic heart disease | 0.146 (0.016-1.355) | 0.091 | 0.310 (0.026-3.631) | 0.351 |
| Previous cerebrovascular event | 0.240 (0.043-1.328) | 0.102 | 0.182 (0.018-1.818) | 0.147 |
| Pre-admission mRS | 0.564 (0.218-1.459) | 0.238 | 0.732 (0.246-2.179) | 0.575 |
| Presence of perfusion asymmetry at any map | 0.144 (0.042-0.494) | 0.002* | 0.117 (0.022-0.611) | 0.011* |
|  |  |  |  |  |
| * Statistically significant data (p < 0.05).  Key: mRS: modified Rankin Scale. | | | | |
